# Genetic analysis of a novel antioxidant multi-target iron chelator, M30 protecting against chemotherapy-induced alopecia in mice

**DOI:** 10.1186/s12885-019-5323-z

**Published:** 2019-02-13

**Authors:** Young-Cheol Lim, Hyeongi Kim, Sang Moo Lim, Jin Su Kim

**Affiliations:** 1 Division of RI application, Korea Institute of Radiological and Medical Sciences, 75 Nowon-Gil, Gongneung-Dong, Nowon-Gu, Seoul, 01812 Korea; 2 Department of Nuclear Medicine, Korea Institute of Radiological and Medical Sciences, Seoul, Korea; 3 Radiological and Medico-Oncological Sciences, University of Science and Technology (UST), Seoul, Korea; 4 Research support team, ANDIVA Inc., Chuncheon, Korea

**Keywords:** Alopecia, Cyclophosphamide, Chemotherapy, M30, Anti-oxidant, Microarray

## Abstract

**Background:**

Chemotherapy-induced alopecia has been well documented as a cause of distress to patients undergoing cancer treatment. Almost all traditional chemotherapeutic agents cause severe alopecia. Despite advances in the treatment of chemotherapy-induced alopecia, there is no effective treatment for preventing chemotherapy-induced alopecia.

**Methods:**

In the present study, we investigated the potential role of a multi-target iron chelator, M30 in protecting against cyclophosphamide-induced alopecia in C57BL/6 mice implanted with an osmotic pump. M30 enhanced hair growth and prevented cyclophosphamide-induced abnormal hair in the mice. Furthermore, we examined the gene expression profiles derived from skin biopsy specimens of normal mice, cyclophosphamide-treated mice, and cyclophosphamide treated mice with M30 supplement.

**Results:**

The top genes namely *Tnfrsf19, Ercc2*, *Lama5*, *Ctsl*, and *Per1* were identified by microarray analysis. These genes were found to be involved in the biological processes of hair cycle, hair cycle phase, hair cycle process, hair follicle development, hair follicle maturation, hair follicle morphogenesis, regulation of hair cycle.

**Conclusion:**

Our study demonstrates that M30 treatment is a promising therapy for cyclophosphamide-induced alopecia and suggests that the top five genes have unique preventive effects in cyclophosphamide-induced transformation.

## Background

Alopecia (hair loss) is a common side effect of systemic cancer treatment using almost all traditional cytostatic chemotherapeutic agents (cyclophosphamide (CTX), doxorubicin, paclitaxel, etoposide), and it is often considered an inevitable consequence of chemotherapy. However, chemotherapy-induced alopecia (CIA) has a negative impact on the wellbeing of many cancer patients [1–4]. In addition, these patients often receive little more counseling than the advice to purchase a wig or other head covering during their cancer treatment [1, 5].

A number of procedures and reagents have been used to ameliorate the side effects of CIA. These include scalp tourniquets, scalp hypothermia, and treatments with minoxidil, AS101, or vitamin D [5–10]. Despite significant advances and efforts in research and development of CIA, no effective and reliable treatment has become available [11, 12], and the investigations have focused on chemotherapy-induced apoptosis and blockade of proliferation [10, 13, 14]. Thus, there remains a need for novel therapies for cancer patients suffering with hair loss. The development of new therapies would be facilitated by understanding the molecular mechanisms of hair loss in CIA.

The N-acetylcysteine (NAC) is an analog and a precursor of glutathione and is known to have a strong antioxidant activity owing to its ability to enhance glutathione synthesis as well as act as an oxygen radical scavenger. NAC protected against doxorubicin-induced alopecia in mice [15], and CTX-induced alopecia in rats [16]. The combination of NAC and a biological response modifier, (ImuVert) induced protection against CIA by a combination of CTX and cytarabine (Ara C, cytosine arabinoside) in neonatal rat models [16]. The observation that antioxidants such as NAC protect against CIA in animals suggests the involvement of reactive oxygen species (ROS) in CIA. However, the mechanism by which ROS induces or promotes CIA has not been investigated [17].

The multi-target iron chelator, M30 [5-(N-methyl-N-propargylaminomethyl)-8-hydroxyquinoline] is a novel antioxidant and protective agent against oxidative stress in a spectrum of diseases. M30 was developed and studied for the prevention and treatment of Alzheimer’s disease, Parkinson’s disease, and other neurodegenerative diseases, which included several therapeutic strategies, such as selective inhibition of monoamine oxidase-B, iron chelation, and anti-oxidation [18–20]. However, there is no report regarding its role in CIA.

In the present study, we aimed to determine the efficacy of M30 against CTX-induced alopecia. Since oxidative stress is one of the major pathological events during the progression of CIA, we hypothesized that M30 exert a beneficial effect against CTX-induced alopecia by inhibiting oxidative stress. We investigated the potential of M30 to stimulate hair re-growth during CTX-induced alopecia using depilated C57BL/6 mice. We further examined the gene expression profiles derived from skin biopsy specimens of depilated normal mice (Normal), CTX-treated depilated mice (CTX), and CTX-treated depilated mice continuously supplemented with M30 (MC). We then assembled a database of publicly available skin microarray samples representing CTX/normal, MC/CTX, and MC/normal groups. We now report that five genes were differentially expressed in three microarray data sets from three different models and these genes were involved in hair cycle and hair follicle responses. These data provide an important foundation for further research into the identification of mechanisms that trigger hair follicle response in order to development of effective approaches for the management of hair loss induced by chemotherapy.

## Methods

### Drugs

CTX and the antioxidant, M30 were purchased as injectable commercial products (Sigma-Aldrich, USA). CTX and M30 were freshly dissolved in phosphate buffer saline (PBS; Gibco, USA) before injection.

### Animals

Six-weeks-old female C57BL/6 mice were obtained from Orient Bio Inc. (Korea). The mice were maintained in temperature-controlled clean racks with a 12-h light/dark cycle. The mice were allowed to acclimatize for 1 week before the start of the experiment. All experiments were performed in accordance with the institutional guidelines of the Korea Institute of Radiological & Medical Sciences (KIRAMS).

### CTX-induced alopecia model

The mice were depilated by shaving, followed by waxing using wax strips (Veet, USA); the skin was washed with PBS before experiments. At 9 days after depilation, the alopecia models were generated by a single intraperitoneal injection of CTX (120 mg/kg body weight) that was freshly prepared in PBS as previously described [21]. Before intraperitoneal injection of CTX, one group of mice (*n* = 5) was subcutaneously implanted with Alzet mini-osmotic pump (model 2004, DURECT, Cupertino, CA) containing M30 in PBS with a delivery rate of 1 mg/d/kg. The other normal (n = 5) and CTX treatment groups (n = 5) underwent sham operations without M30 supplement.

### Preparation of tissue samples and observation of hair follicles

For the euthanization of mice, CO_2_ was injected at a rate of 10–30% per minute, that gradually fills the euthanasia chamber. Euthanization was performed by trained member. Then full-skin thickness samples of skin tissue were excised using scissors and immediately fixed using 4% paraformaldehyde in PBS for overnight at 4 °C. After fixation, the samples were incubated with 30% sucrose in PBS for 24 h at 4 °C. Then, the skin wasr embedded in O.C.T compound (Fisher Scientific, Pittsburg, USA) and frozen in liquid nitrogen. After freezing, the samples were stored in liquid nitrogen until further processing. Each specimen was sliced into 5 μm thickness section and observed by confocal microscopy. For microarray analysis, skin tissues from the mice were excised and immediately stored in liquid nitrogen in cryotubes until microarray analysis.

### Target labeling and hybridization to microarray

For each RNA sample, synthesis of the target cRNA probes and hybridization were performed using Agilent’s Low Input QuickAmp Labeling Kit (Agilent Technologies, USA) according to the manufacturer’s instructions. Briefly, each 25 ng total RNA and the T7 promoter primer were mixed and incubated at 65 °C for 10 min. The cDNA master mix (5x First strand buffer, 0.1 M DTT, 10 mM dNTP mix, RNase-Out, and MMLV-RT) was prepared and added to the reaction mixer. The samples were incubated at 40 °C for 2 h, and then dsDNA synthesis was terminated by incubating at 70 °C for 10 min. The transcription master mix was prepared as the manufacturer’s protocol (4x Transcription buffer, 0.1 M DTT, NTP mix, 50% PEG, RNase-Out, Inorganic pyrophosphatase, T7-RNA polymerase, and Cyanine 3-CTP). Transcription of the dsDNA was preformed by adding the transcription master mix to the dsDNA reaction samples and incubating at 40 °C for 2 h. Amplified and labeled cRNA was purified on an RNase mini column (Qiagen) according to the manufacturer’s protocol. The Labeled cRNA target was quantified using an ND-1000 spectrophotometer (NanoDrop Technologies, USA). After verifying labeling efficiency, each 1650 ng of cyanine 3-labeled cRNA target was carried out the fragmentation of cRNA was fragmented by adding 10x blocking agent and 25x fragmentation buffer and incubating at 60 °C for 30 min. The fragmented cRNA was resuspended with 2x hybridization buffer and directly pipetted onto the assembled Agilent Mouse (V2) Gene Expression 4 × 44 K Microarray. The arrays were hybridized at 65 °C for 17 h using hybridization oven (Agilent Technologies, USA). The hybridized microarrays were washed as indicated in the manufacturer’s washing protocol (Agilent Technologies, USA).

### Data acquisition and analysis

The hybridization images were analyzed by Agilent DNA Microarray Scanner (Agilent Technologies, USA) and data quantification was performed using Agilent Feature Extraction software 10.7 (Agilent Technologies, USA). The average fluorescence intensity of each spot was calculated and the local background was subtracted. Normalization of data and selection of fold-changed genes were performed using GeneSpringGX 7.3.1 (Agilent Technologies, USA). Normalization for Agilent one-color method was performed, which is Data transformation: Set measurements less than 5.0 to 5.0 and Per Chip: Normalize to 50th percentage. Each average normalized ratio was calculated by dividing the average of the control normalized signal intensity by the average of the test normalized signal intensity. Functional annotation of the genes was performed according to Gene Ontology™ (GO) Consortium (http://www.geneontology.org/index.shtml) by GeneSpringGX 7.3.1.

### mRNA quantification by quantitative reverse transcription PCR (qRT-PCR)

Total RNA was extracted from mouse skin by using TRIzol reagent (Invitrogen, USA). Reverse transcription was performed on total RNA using SuperScript II reverse transcriptase (Invitrogen, USA) according to the manufacturer’s instructions. The resulting cDNA was amplified using the following primer pairs: (5′ → 3′) Tnfrsf19 (Forward: ATGACAGGGATGATCAAAGC, Reverse: TCGGCATGTGGAAAATATCT), Ercc2 (Forward: AAGAGGAGCCCAAAAAGACA, Reverse: CATCCGTGACATCAGTCAGA), Lama5 (Forward: TGCTTGAGGAAGCTGCTGAT, Reverse: CACTGCCCCCTGGATTTGTA), Ctsl (Forward: GGGACAACCACTGTGGACTT, Reverse: CTCATTACCGCTACCCATCA), Per1 (Forward: TGCATCGTCCCATTGTGAGT, Reverse: CCATGCCAGCCTGGATACTT), GAPDH (Forward: GGCATTGCTCTCAATGACAA, Reverse: ATGRAGGCCATGAGGTCCAC). Real-time PCR was performed on the StepOnePlus™ Real Time PCR System (Applied Biosystems, USA) using the SYBR Green PCR Kit (Applied Biosystems, USA), according to the manufacturer’s instructions. The thermal cycling conditions were 95 °C for 10 min followed by 40 cycles of 95 °C for 15 s and at optimal Tm (59 °C) for 30 s. The data were analyzed using the StepOne software v2.2.2 (Applied Biosystems, USA). The expression levels of each mRNAs were normalized to the endogenous control GAPDH and were calculated using the 2-ΔΔCt method.

### Gene network construction and visualization

The BisoGenet plug-in [22] from Cytoscape software version 2.7.0 [23] was used to build and visualize the networks for the top five significant genes using the respective list of significant genes of the GO categories. All available data sources in BisoGenet (including BIOGRID, DIP, BIND, and others) were selected to generate the interactions.

### Statistical analysis

Data processing was performed using Origin 6.1 (OriginLab, Northampton, MA). Statistical significance was determined using Student’s t-tests and ANOVA. A *p-*value less than 0.05 was considered statistically significant.

## Results

### M30 prevents CTX-induced alopecia in C57BL/6 mice

**Fig. 1:**
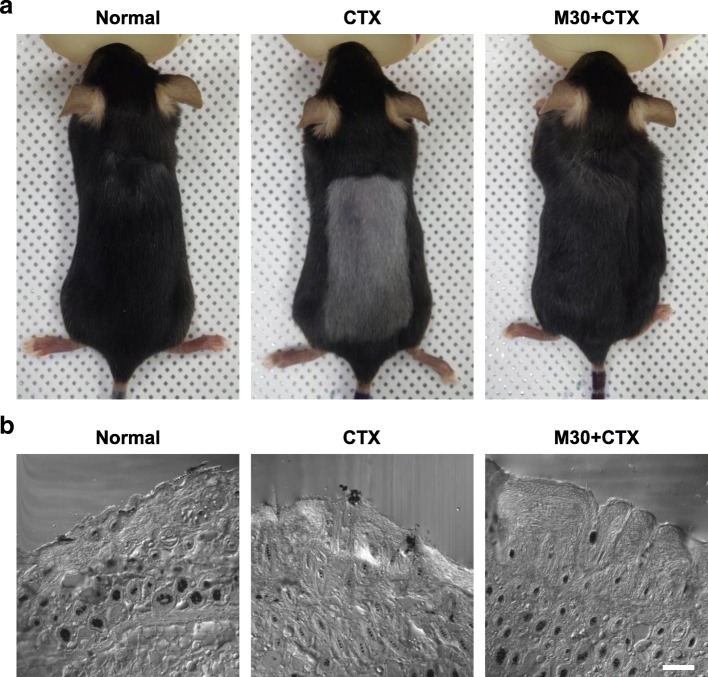
Macroscopic and histological effects of M30 on CTX-induced alopecia in C57BL/6 mice. The mice were depilated using wax strips and intraperitoneally injected with CTX with or without M30 supplement (see Methods section). After CTX treatment, shaved skin of the normal, CTX-treated, and M30-supplemented CTX-treated mice was photographically observed 2 weeks after CTX treatment. **a** Representative images of dorsal skin of normal mice (Normal), CTX-treated mice (CTX), and M30-supplemented CTX-treated mice (M30 + CTX) are shown. The sample tissue was sliced into 5-μm-thick transverse sections and these sections (*n* = 5) were observed using a confocal microscope. **b** Representative images of skin of normal mice (Normal), CTX-treated mice (CTX), and M30-supplemented CTX-treated mice (M30 + CTX) are shown. Scale bar = 100 μm

To investigate whether M30 could prevent CTX-induced alopecia in mice, M30 was continuously infused using subcutaneously implanted osmotic pumps in depilated C57BL/6 mice. The hair was depilated on the dorsal surface of the normal and M30-supplemented mice, and then alopecia was induced by a single intraperitoneal injection of CTX (120 mg/kg body weight). The shaved skin of the telogen mice was pink and darkened with anagen initiation. Two weeks after injection of CTX, normal black hair was apparent in the normal mice, whereas abnormal gray hair was apparent in the CTX-treated mice. However, M30 supplement prevented the growth of abnormal gray hair in the CTX-treated mice (Fig. 1a). To further study the M30 normalized abnormal gray hair; we analyzed transverse sections of the dorsal skin from 2 weeks after injection of CTX. As suggested by the microscopic observation, M30 markedly increased the depth and size of the hair follicles, prevented the dystrophic changes seen with the CTX-treated mice, and normalized the appearance of the skin to that of normal mice (Fig. 1b).

### Differential gene expression by CTX and M30

**Fig. 2:**
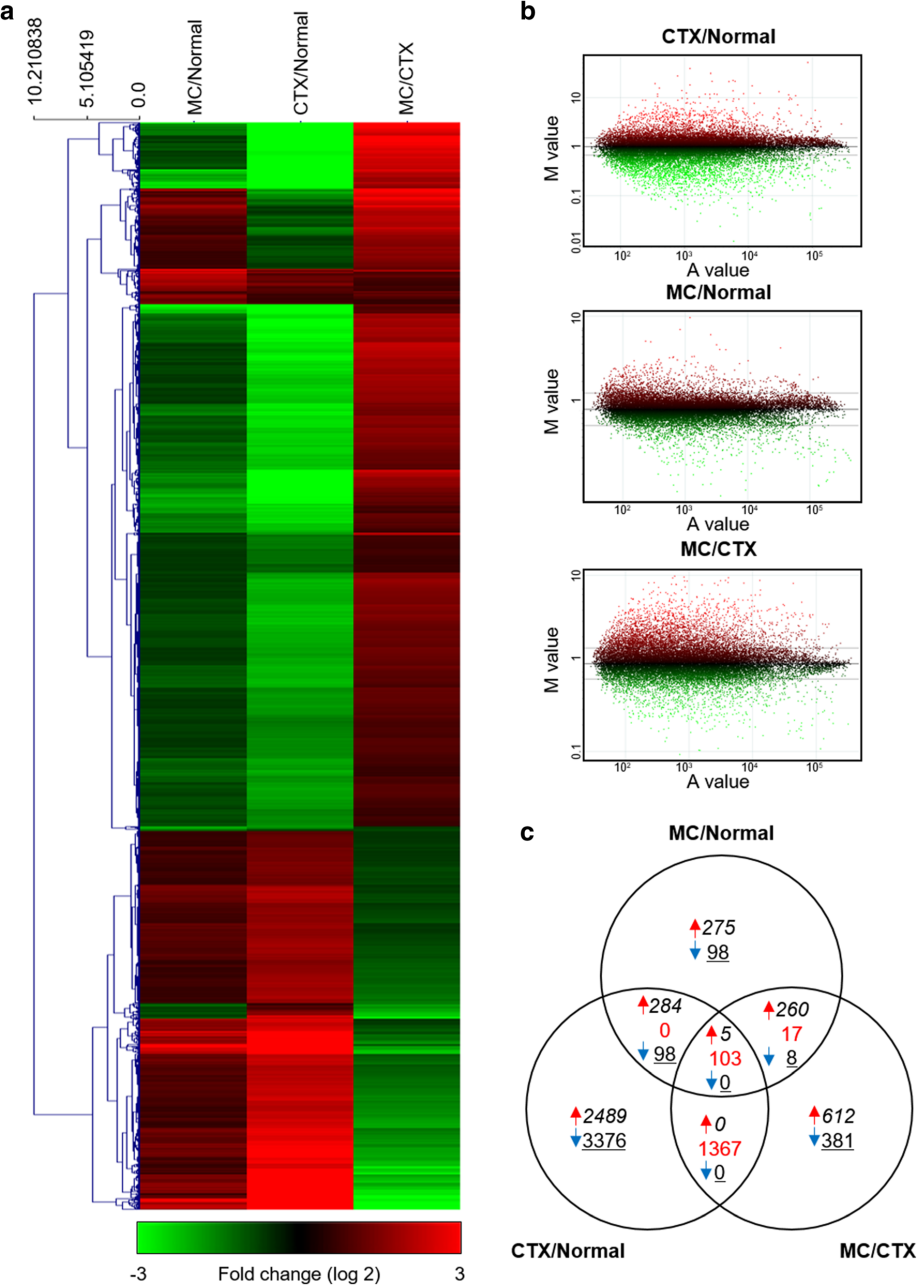
Representative MA plot of changes and hierarchical clustering analyses in gene expression levels after CTX and M30 treatment. **a** Hierarchical cluster analysis of all samples in the gene expression microarray. Genes that were upregulated relative to control are shown in red and those that were downregulated are shown in green. The expression levels of these genes were altered ≥1.5-fold or ≤ 0.666-fold in the CTX/Normal, MC/CTX, and MC/Normal condition (*p* < 0.05). **b** MA plots comparing each of the three data sets for a representative sample. Gene expression profiles from the normal mouse skin compared with CTX-treated mouse skin (CTX/Normal), normal mouse skin compared with M30-supplemented CTX-treated mouse skin (MC/CTX), and CTX-treated mouse skin compared with M30-supplemented CTX-treated mouse skin (MC/Normal). MA plot where M = log (Cy5/Cy3) is the log ratio of the two dyes used in the hybridization, and A = [log (Cy5) + log (Cy3)]/2 is the average of the log intensities. **c** Venn diagram showing the number of genes regulated by CTX, or CTX with M30. The number of upregulated, contra-regulated, and downregulated genes that responded commonly or uniquely to the treatments is shown in red arrows, red texture, and blue arrows, respectively

To investigate the alterations of gene expression in mouse skin during CTX and M30 treatment, we isolated total RNA from the skin of normal, CTX-treated mice, and M30 supplemented CTX-treated and we applied this RNA to the assembled Agilent Mouse (V2) Gene Expression 4 × 44 K Microarray, which contains 39,429 mouse genes. After hybridization, the microarray slide was scanned and analyzed. Each mouse gene was quantified according to its Cy3-labeled versus Cy5-labeled signal intensity. The graphs are shown (Fig. 2a) on a log scale using the Agilent Feature Extraction software 10.7 (Agilent Technologies, USA). These raw images were normalized by MA plot using locally weighted scatter plot smoothing (LOWESS) method. We applied the MA plot normalization process where M = log (Cy5/Cy3) is the log ratio of the two dyes used in the hybridization, and A = [log (Cy5) + log (Cy3)]/2 is the average of the log intensities. A skewed form of ratio pattern before normalization changed to a linear pattern centered on zero after normalization. To understand which gene expression had changed, hierarchical clustering analysis was performed by using 9373 genes as shown in Fig. 2b.

### Hierarchical cluster analysis

Differences in the expression patterns of the protein-coding genes in the dorsal skin among normal mice, CTX-treated mice, and M30 supplemented CTX-treated mice were analyzed. By analyzing a genome-wide microarray, we observed significant transcriptional changes in mouse skin. We compared the CTX-treated mice with normal mice (CTX/Normal), M30 supplemented CTX-treated mice with normal mice (MC/Normal), and M30 supplemented CTX-treated mice with CTX-treated mice (MC/CTX), using a filter criterion of > 1.5-fold change with *p* < 0.05. We then compared the results of the skin samples obtained from these groups using the hierarchical clustering method in Cluster 3.0 software. The up-regulated genes were highly expressed in the experimental group (red), and the expression levels of the down-regulated genes were significantly decreased (green) (Fig. 2b). The cluster analysis shows that CTX and M30 induce differences in gene expression in the skin of the mice, and the stylized Venn diagram depicts the patterns of changes in the gene expression levels in each skin sample. The numbers of up-regulated, contra-regulated, and down-regulated genes that responded commonly or uniquely in response to the treatment are shown with red arrows, red texture, and blue arrows, respectively (Fig. 2c).

### Results of the differentially expressed gene analysis

Based on Fig. 2b, we screened out 7722 genes (3422 up-regulated and 4300 down-regulated genes) in CTX/Normal, 1148 genes (866 up-regulated and 282 down-regulated genes) in MC/Normal, and 2753 genes (1715 up-regulated and 1038 down-regulated genes) in MC/CTX, using a filter criterion at least 1.5-fold change with *p* < 0.05. In addition, we rescreened recovered genes by M30 treatment against the CTX-regulated genes. The M30-down-regulated 644 genes against up-regulation by CTX were selected using a filter criterion of greater than 1.5-fold change (CTX/normal) and less than 0.666-fold change (MC/CTX) with p < 0.05. Moreover, the M30-up-regulated 241 genes against down-regulation by CTX were rescreened out using a filter criterion of less than 0.666-fold change (CTX/normal) and greater than 1.5-fold change (MC/CTX) with *p* < 0.05 (Table 1).

**Table 1:** Number of regulated genes in skin of mice 2 weeks after administration of CTX with or without M30

Condition	Fold-change	*P* value	Mice (n)	Regulated genes (no.)		
				Total	Up	Down
CTX/Normal	> 1.5	< 0.05	Normal^a^ (3), CTX^b^ (3)	7722	3422	4300
MC/Normal	> 1.5	< 0.05	Normal (3), MC^c^ (3)	1148	866	282
MC/CTX	> 1.5	< 0.05	CTX (3), MC (3)	2753	1715	1038
Condition 1	> 1.5 (CTX/Normal) & < 0.666 (MC/CTX)	< 0.05	Normal (3), CTX (3), MC (3)	644		
Condition 2	< 0.666 (CTX/Normal) & > 1.5 (MC/CTX)	< 0.05	Normal (3), CTX (3), MC (3)	241		

^a^Normal is normal mice: ^b^CTX is cyclophosphamide-treated mice: ^c^MC is M30-supplemented cyclophosphamide-treated mice

### Gene ontology-based analysis

**Fig. 3:**
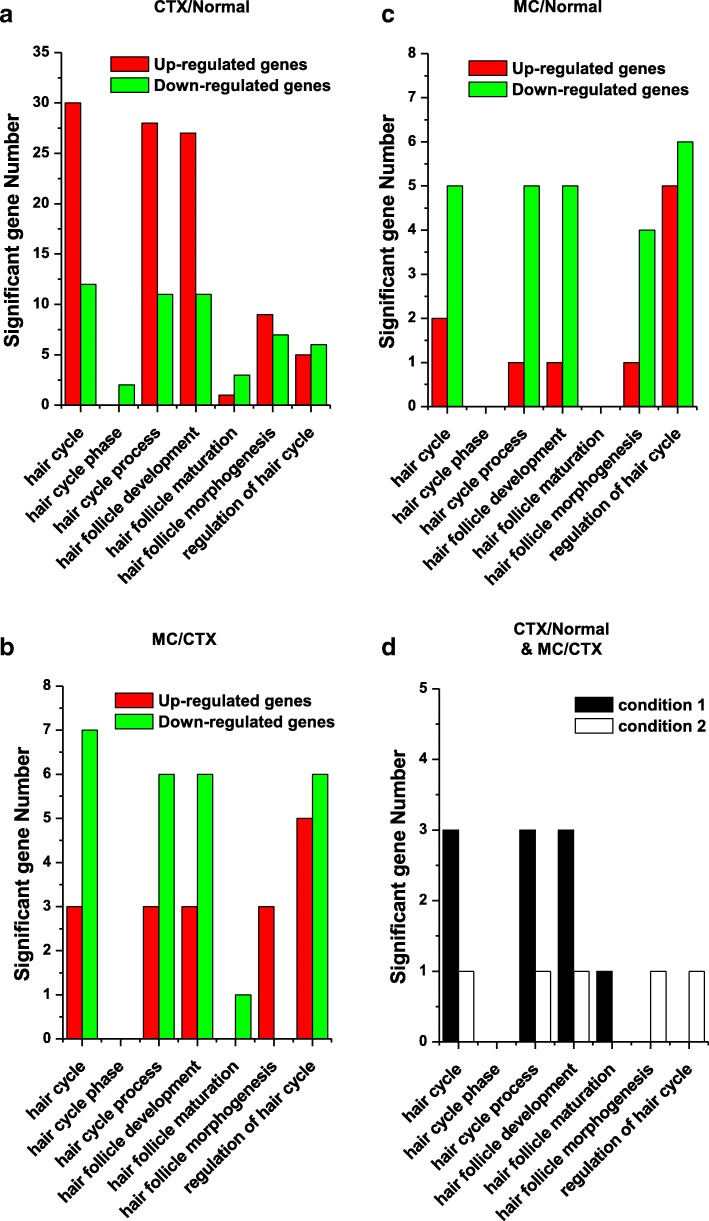
The main functional categories showing significantly changed hair-related genes after CTX and M30 treatment. **a**-**d** The number of differentially expressed genes that were demonstrated in the following GO terms is indicated: hair cycle, hair cycle phase, hair cycle process, hair follicle development, hair follicle maturation, hair follicle morphogenesis, and regulation of hair cycle. Upregulated or downregulated genes in the following conditions of **a** CTX/Normal, **b** MC/CTX, and **c** MC/Normal are shown. **d** M30-recovered genes against CTX treatment in conditions 1 and 2 (see Table 2) are shown

In further study, the functional annotation of the genes was assessed using a GO based biological property analysis (QuickGO; https://www.ebi.ac.uk/QuickGO/). The numbers of interesting genes were categorized as those being involved in the hair cycle, hair cycle phase, hair cycle process, hair follicle development, hair follicle maturation, hair follicle morphogenesis, and regulation of hair cycle in CTX/Normal, MC/CTX, and MC/Normal conditions, (Fig. 3a-c). Among these, the top five target genes, namely *Tnfrsf19, Ercc2, Lama5,* Ctsl, and Per1 were screened and the data are presented in Fig. 3d and Table 2. Tnfrsf19, Ercc2, Lama5, and Ctsl, are associated with hair cycle, hair cycle process, and hair follicle development. And Per1, Ercc2, and Ctsl*,* are associated with regulation of hair cycle, hair follicle maturation, and hair follicle morphogenesis, respectively (Table 2). Taken together, these results show that CTX induces the differential expression of hair cycle, and hair follicle associated genes, which were recovered by M30 treatment.

**Table 2:** The five recurrently hair-related regulated genes after CTX and M30 treatment

Group	Gene symbol	Common name	GO annotation						
			Hair cycle	Hair cycle phase	Hair cycle process	Regulation of hair cycle	Hair follicle development	Hair follicle maturation	Hair follicle morphogenesis
Condition 1	Tnfrsf19	Tumor necrosis factor receptor superfamily member 19	Yes		Yes		Yes		
	Ercc2	Excision Repair Cross-Complementation Group 2	Yes		Yes		Yes	Yes	
	Lama5	Laminin, Alpha 5	Yes		Yes		Yes		
Condition 2	Ctsl	Cathepsin L	Yes		Yes		Yes		Yes
	Per1	Period Circadian Clock 1				Yes			

### Validation of microarray findings with quantitative RT-PCR (qRT-PCR)

To validate the microarray results, we quantified the expression of five target genes by quantitative RT-PCR (qRT-PCR) in normal, CTX, and MC sample. All qRT-PCR analyses were performed in samples previously used for the microarray experiments. Table 3 summarize the gene expression measurements of the five validated genes by qRT-PCR. We found that both methods (microarray analysis and qRT-PCR) detected similar patterns for the five target genes by condition 1 and condition 2. The respective *p*-values for the microarray and qRT-PCR data were significant at the 0.05 level (Table 3).

**Table 3:** Quantitative RT-PCR validation of microarray data

Group	Gene symbol	Common name	Microarray (fold)		qRT-PCR (fold)		Microarray vs qRT-PCR(*p*-value)	
			CTX^b^/ Normal^a^	MC^c^/CTX	CTX /Normal	MC/CTX	CTX /Normal	MC/CTX
Condition 1	Tnfrsf19	Tumor necrosis factor receptor superfamily member 19	1.651	0.579	17.882	0.461	0.0058	0.431
	Ercc2	Excision Repair Cross-Complementation Group 2	1.596	0.660	10.660	0.765	0.022	0.216
	Lama5	Laminin, Alpha 5	2.033	0.443	7.160	0.478	0.062	0.654
Condition 2	Ctsl	Cathepsin L	0.588	2.119	0.620	1.758	0.884	0.367
	Per1	Period Circadian Clock 1	0.333	2.586	0.730	1.260	0.133	0.119

^a^Normal is normal mice: ^b^CTX is cyclophosphamide-treated mice: ^c^MC is M30-supplemented cyclophosphamide-treated mice

**Table 4:** The alterated genes after CTX and M30 treatment

Angiogenesis			Aging			Cell proliferation			Cell migration		
Gene symbol	Fold change		Gene symbol	Fold change		Gene symbol	Fold change		Gene symbol	Fold change	
Condition 1*	CTX/Normal	MC/CTX	Condition 1	CTX/Normal	MC/CTX	Condition 1	CTX/Normal	MC/CTX	Condition 1	CTX/Normal	MC/CTX
Shh	11.540	0.384	Gjb6	9.359	0.258	Shh	11.540	0.384	S100a8	11.647	0.555
Lef1	9.036	0.201	Krt25	5.496	0.139	Oca2	11.515	0.072	Shh	11.540	0.384
Adm2	7.801	0.213	Alox12	4.174	0.352	Lef1	10.260	0.184	Saa3	11.365	0.268
Robo1	4.914	0.418	Slc34a2	3.581	0.656	Tspan1	10.093	0.210	Foxe1	11.226	0.221
E2f8	4.537	0.307	H2afx	3.312	0.422	Lef1	9.036	0.201	Lef1	10.260	0.184
Condition 2*	CTX/Normal	MC/CTX	Condition 2	CTX/Normal	MC/CTX	Condition 2	CTX/Normal	MC/CTX	Condition 1	CTX/Normal	MC/CTX
Tbx4	0.167	4.913	Ifi27l2a	0.165	1.675	Dmbt1	0.122	3.491	Ccl6	0.079	3.826
Spi1	0.163	3.851	Ccl2	0.137	2.583	Slc11a1	0.107	5.273	Ccl7	0.073	4.394
Nov	0.162	4.204	Il6	0.086	4.170	Enpep	0.103	8.165	Retnlg	0.047	21.223
Ccl2	0.137	2.583	Pot1b	0.067	2.010	Gapt	0.086	5.105	Ccl24	0.043	8.652
Enpep	0.103	8.165	Fos	0.052	4.141	Il6	0.086	4.170	Cxcl1	0.035	4.222
Cell death			Apoptosis			Inflammatory response			RNA splicing		
Gene symbol	Fold change		Gene symbol	Fold change		Gene symbol	Fold change		Gene symbol	Fold change	
Condition 1	CTX/Normal	MC/CTX	Condition 1	CTX/Normal	MC/CTX	Condition 1	CTX/Normal	MC/CTX	Condition 1	CTX/Normal	MC/CTX
S100a8	11.647	0.555	S100a8	11.647	0.555	S100a8	11.647	0.555	Snrpc	4.166	0.564
Shh	11.540	0.384	Shh	11.540	0.384	Saa3	11.365	0.268	Rbfox3	3.293	0.317
Avp	10.850	0.317	Avp	10.850	0.317	Camp	4.323	2.691	Ppil1	2.778	0.480
Lef1	10.260	0.184	Lef1	10.260	0.184	Olr1	3.809	0.515	Ddx39	2.238	0.551
Gjb6	9.359	0.258	Gjb6	9.359	0.258	Crhbp	3.421	0.434	Rbmx	2.146	0.511
Condition 2	CTX/Normal	MC/CTX	Condition 2	CTX/Normal	MC/CTX	Condition 2	CTX/Normal	MC/CTX	Condition 2	CTX/Normal	MC/CTX
Ptgis	0.141	5.335	Ptgis	0.141	5.335	Pf4	0.084	5.956	Snrpn	0.527	1.992
Gzma	0.139	8.220	Gzma	0.139	8.220	Ccr5	0.083	5.403	Rbpms	0.503	1.687
Scn2a1	0.116	5.697	Scn2a1	0.116	5.697	Ccl7	0.073	4.394	Celf2	0.493	1.719
Il6	0.086	4.170	Il6	0.086	4.170	Ccl24	0.043	8.652	Rbfox1	0.211	3.440
Cd5l	0.059	3.309	Cd5l	0.059	3.309	Cxcl1	0.035	4.222	Nova1	0.171	3.988
Extracellular matrix			Immune response			Secretion					
Gene symbol	Fold change		Gene symbol	Fold change		Gene symbol	Fold change				
Condition 1	CTX/Normal	MC/CTX	Condition 1	CTX/Normal	MC/CTX	Condition 1	CTX/Normal	MC/CTX			
Col10a1	27.343	0.043	S100a8	11.647	0.555	Crhr1	15.534	0.178			
Shh	11.540	0.384	Lef1	10.260	0.184	Trim9	11.777	0.246			
Nav2	7.185	0.283	Exo1	5.591	0.251	Crhr1	8.741	0.170			
Gpc5	3.730	0.549	Srms	4.538	0.452	Nkd2	5.288	0.463			
Wnt5a	3.689	0.531	Itgal	4.378	0.482	Syt7	5.101	0.270			
Condition 2	CTX/Normal	MC/CTX	Condition 2	CTX/Normal	MC/CTX	Condition 2	CTX/Normal	MC/CTX			
Dmbt1	0.122	3.491	C1qa	0.057	8.083	Kcnma1	0.151	3.865			
Cfp	0.118	2.918	Oas3	0.051	3.151	Fcer1g	0.118	3.346			
Myoc	0.081	9.790	Ccl24	0.043	8.652	Fcgr3	0.105	6.135			
Mamdc2	0.076	9.511	Fcna	0.041	12.585	Agtr2	0.092	3.212			
Mmp11	0.070	8.145	Cxcl1	0.035	4.222	Il6	0.086	4.170			

*Condition 1 & 2 (*p* < 0.05)

### Gene network analyses

**Fig. 4:**
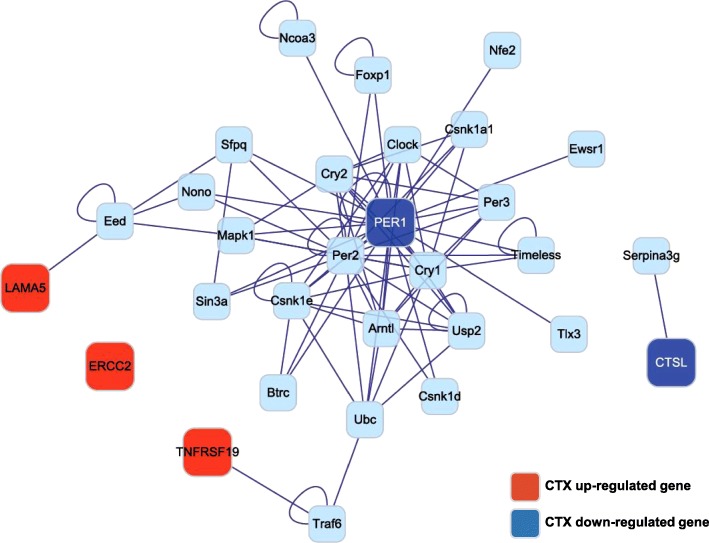
Interaction networks of five significantly changed hair-related genes after CTX and M30 treatment. Five target gene interaction networks were constructed by Cytoscape software (version 2.7.0; http://www.cytoscape.org). The top five target genes were screened according to the rank of target gene pair-specific context score. The genes in red nodes indicate CTX upregulated genes, blue nodes indicate downregulated genes, and the genes in edges indicate the interactive 25 genes

Following combined target prediction, overlapped gene deletion and validation, five genes were identified as targeted by M30 treatment, as shown in Fig. 3d and Table 2. To gain insight into the dynamics of these five significant genes and associated genes, we mapped the gene interactions network based on datasets derived from Proteomics or Genomics experiments. The five genes and their associated 25 genes are displayed in the gene network. The CTX up-regulated genes (*Tnfrsf19*, *Ercc2*, and *Lama5*) were shown in red nodes and down-regulated genes (*Ctsl* and *Per1*) in blue nodes, with 25 genes in the edges, respectively (Fig. 4). The entire network were verified for interactive visualization of gene interaction networks in the Cytoscape session data. The network can be loaded and visualized using Cytoscape (refer to the Methods section).

## Discussion

During the last several decades, clinicians have attempted to develop nonpharmacological and pharmacological therapies to prevent alopecia from chemotherapy. A mechanical strategy, the scalp tourniquet has been applied in the past. The inflatable scalp tourniquet reduces blood supply to the scalp and hair follicles during chemotherapy in patients for prevention of CIA [7, 24]. However, concern has been expressed that this scalp cooling method promotes vasoconstriction to the scalp and inhibits temperature-dependent uptake of chemotherapeutic drugs in the hair follicle. In addition, pharmacologic strategies against CIA also have been applied to promote hair growth and prevent hair loss. The antioxidant, minoxidil is well known to promote hair growth in male-pattern baldness, and a local injection of minoxidil protected against cytarabine-induced alopecia [25], but not cyclophosphamide-induced alopecia in a neonatal rat model [26]. In previous studies, the various therapies have not provided any evidence of hair-loss prevention, and the effects of certain agents were dependent on the model being used [11].

The aim of this study was to investigate the ability of a novel antioxidant multi-target iron chelator M30, to promote hair growth in CTX-induced alopecia. In addition, we investigated the pathogenesis of CTX-induced alopecia and M30 protection against CTX-induced alopecia by comparing the gene expression profiles. In this study, we used C57BL/6 mice for CTX-induced alopecia models and global microarray profiling to study changes in the gene expression signature of mouse skin during CTX treatment with or without M30 treatment. We then compared the related gene expression profiles of the treated mice to those of the normal mice. CTX is responsible for several skin damages.

Our results demonstrate the preventive role of M30 against CTX-induced alopecia in C57BL/6 mice. M30 supplement therapy has been shown previously to improve cognitive impairment and reduce Alzheimer’s-like neuropathology in mouse models of Alzheimer’s disease [27, 28], but not CTX-induced alopecia. We systemically administered M30 using osmotic pumps to C57BL/6 mice to investigate the protective role of M30 against CTX-induced alopecia. The most important finding of our study was that M30-treated mice showed normal hair growth on the depilated skin of mice (Fig. 1).

We further compared the molecular signature of CTX-treated mouse skin with and without M30 treatment with the skin of normal mice using global microarray profiling. cRNA microarray technology has become a widely used application for molecular profiling, and the techniques used to analyze the extensive quantity of data generated are variable. A hierarchical clustering heat map (Fig. 2b) and stylized Venn diagram (Fig. 2c) depict the patterns of changes in the gene expression levels in each sample. A detailed list of the gene signatures is presented in Table 2. We were interested in the general pattern of expression in the skin, changes in response to CTX treatment and the prevention of CTX induced increased or decreased gene expression by M30.

Our study is the first to introduce the concept of systemic study by CTX and M30. By analyzing a genome-wide microarray, we observed significant transcriptional changes in 7722 genes of the CTX-treated skin when compared with that of normal skin using a filter criterion of least 1.5-fold change with *p* < 0.05. Approximately 3422 genes were upregulated and 4300 genes were downregulated by CTX treatment. We further observed that M30 recovered genes against CTX transcriptional upregulation and downregulation. The 644 and 241 genes that were altered by CTX treatment but recovered by M30 treatment are shown in Table 1. These results indicate that M30 has preventive activities against CTX-induced pathological changes in the skin.

**Fig. 5:**
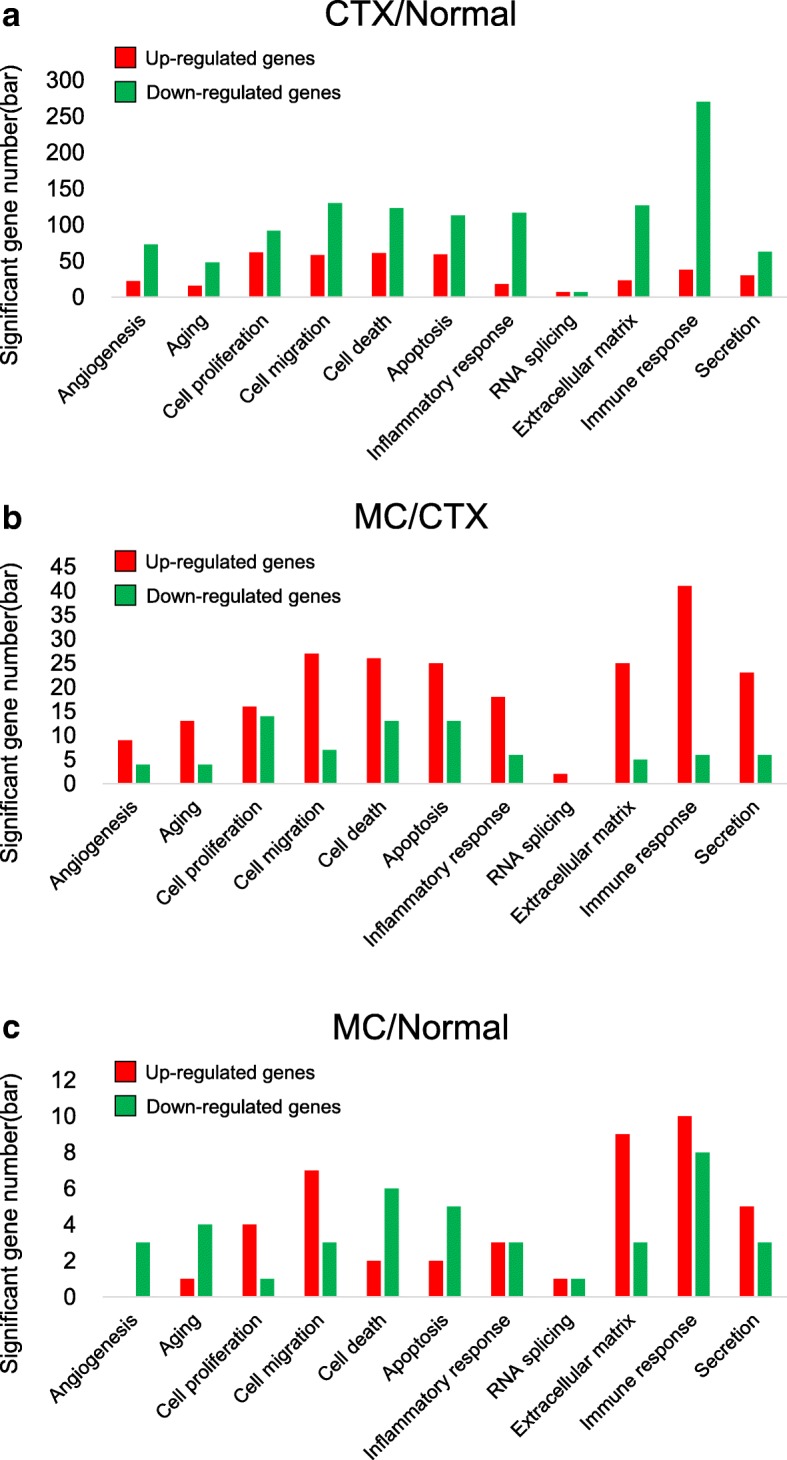
The main functional categories showing significantly changed genes after CTX and M30 treatment. **a**-**c** The number of differentially expressed genes that were demonstrated in the following GO terms is indicated: angiogenesis-, aging-, cell proliferation-, cell migration-, cell death-, apoptosis-, inflammation-, RNA splicing-, extracellular matrix-, immune response-, and secretion-related genes. **a** CTX/Normal, **b** MC/CTX, and **c** MC/Normal

**Fig. 6:**
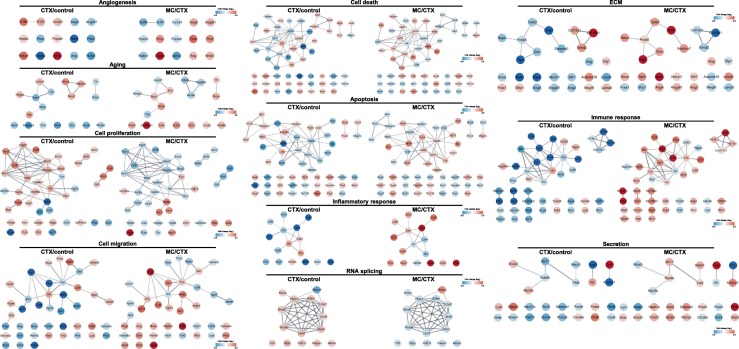
Interactions networks of five significantly changed genes after CTX and M30 treatment. Target gene interaction networks were constructed by Cytoscape software (version 2.7.0; http://www.cytoscape.org). The genes in red nodes indicate CTX upregulated genes, blue nodes indicate downregulated genes, and the genes in edges indicate the interactive 25 genes

Functional annotation of the genes was assessed using GO-based biological property analysis (QuickGO; https://www.ebi.ac.uk/QuickGO/). The interesting genes were categorized as those being involved in the hair cycle, hair cycle phase, hair cycle process, hair follicle development, hair follicle maturation, hair follicle morphogenesis, and regulation of hair cycle (Fig. 3). Then, we validated expression of the five target genes at the transcript level with qRT-PCR data. In addition, we reported that gene interaction networks belonging to different modes such as activation, binding, and post-transcriptional modification, among others, led to the identification of new edges and ultimately contributed to identifying an M30 regulatory network in the CTX-treated skin. The derived network was visualized by Cytoscape (http://www.cytoscape.org/) as shown in Fig. 4. The hair-related GO and network analyses are shown in Figs. 3 and 4. Additionally, the unbiased GO and pathway analyses are shown in Figs. 5, 6 and Table 4.

## Conclusions

These results provide new and useful information to support the epidemiological data showing that M30 replacement is a promising therapeutic strategy for CTX-induced alopecia, and that the top five genes, namely *Tnfrsf19, Ercc2, Lama5, Ctsl*, and *Per1*, might be involved in CTX-induced pathology. Although not directly clinically relevant, our findings suggest that these genes have a unique preventive role in CTX-induced alopecia. However, further studies, including histological analysis of skin specimens, are needed to confirm the results and to verify the protective effect of M30 against CTX treatment.

## Abbreviations

CIA: Chemotherapy-induced alopecia
CTX: Cyclophosphamide
GO: Gene ontology
NAC: N-acetylcysteine
ROS: Reactive oxygen species
